# Supplementary material for: Acquired hypothyroidism, iodine status and hearing impairment in adults: A pilot study
Source: PLoS One. 2025 Jan 30;20(1):e0305787. doi: 10.1371/journal.pone.0305787 (PMC11781629; doi:10.1371/journal.pone.0305787)
Supplement: Attachment — Submitted filename: Review Comments.docx [file pone.0305787.s001.docx]

The manuscript “Acquired hypothyroidism, iodine status and hearing impairment in adults: a pilot study investigate the rate of hearing impairment in acquired hypothyroid patients. Manuscript designed well and consider for publication. However there are few corrections, which need to incorporate in manuscript.

1. Page 6, line no. 148, explain the criteria for selection of controls and mention that Hearing acuity test was performed in control group or not.

2. Page number 7, line number 159, 160 and 161, the authors have mentioned that Overt primary hypothyroidism is defined as thyroid-stimulating hormone (TSH) concentrations above the reference range and free thyroxine (T4) concentrations below the reference range. The diagnosis of overt hypothyroidism was confirmed by a second blood sample. The authors need to mention reference range and diagnosis criteria with appropriate use of reference.

3. Page 8, line no. 189, mention the name of statistical software used for data analysis.

4. Line no. 146-147, Hearing tests were done at the Department of Otorhinolaryngology of the University Hospital Kralovske Vinohrady in Prague. The hearing examination was conducted by trained audiologist or not?
