# Supplementary material for: Acquired hypothyroidism, iodine status and hearing impairment in adults: A pilot study
Source: PLoS One. 2025 Jan 30;20(1):e0305787. doi: 10.1371/journal.pone.0305787 (PMC11781629; doi:10.1371/journal.pone.0305787)
Supplement: Attachment — Submitted filename: Response to Reviewers.docx [file pone.0305787.s002.docx]

Response to Reviewers

First of all, we would like to thank the reviewers for reading and making the comments to our work. We appreciate it very much.

**Reviewers' comments #1:** Study is underpowered with only 30 cases/ controls and cannot answer the research question that hypothyroid patients are more prone to hearing loss than euthyroid controls ( given the anticipated effect size and precision of measurement).
**Response to Reviewer #1:** We did the power analysis. The effect size for discrimination between groups for significance level p<0.05 and power=0.8 was estimated using Mann Whitney test. In our data we were able to differentiate between the groups with effect size 0.385 (to find a moderate effect). We added to our statistics.

**Reviewers' comments #1:** The introduction and discussion are excessively detailed. The results section similarly has extensive information not relevant to the research question.

**Response to Reviewer #1:** We removed some of the excessively detailed parts from the introduction, results and discussion. You are right, we were too excited about too many details. However, we wanted to show the topic of hearing impairment and hypothyroidism comprehensively. There are too many unknown gaps.

**Reviewers' comments #2:**
Page 6, line no. 148, explain the criteria for selection of controls and mention whether the hearing acuity test was performed in the control group or not.

**Response to Reviewer #2:** We explained it in more detail and hope to make it clearer (marked).

**Reviewers' comments #2:** Page number 7, line number 159, 160 and 161, the authors have mentioned that Overt primary hypothyroidism is defined as thyroid-stimulating hormone (TSH) concentrations above the reference range and free thyroxine (T4) concentrations below the reference range. The diagnosis of overt hypothyroidism was confirmed by a second blood sample. The authors need to mention reference range and diagnosis criteria with appropriate use of reference.
**Response to Reviewer #2:** The reference ranges of our lab are mentioned below (marked), we used the appropriate reference.

**Reviewers' comments #2:** Page 8, line no. 189, mention the name of statistical software used for data analysis.
**Response to Reviewer #2:** We added all the softwares.

**Reviewers' comments #2:** Line no. 146-147, Hearing tests were done at the Department of Otorhinolaryngology of the University Hospital Kralovske Vinohrady in Prague. The hearing examination was conducted by trained audiologist or not?

**Response to Reviewer #2:** Yes, by a trained audiologist (marked).
