# Supplementary material for: Acquired hypothyroidism, iodine status and hearing impairment in adults: A pilot study
Source: PLoS One. 2025 Jan 30;20(1):e0305787. doi: 10.1371/journal.pone.0305787 (PMC11781629; doi:10.1371/journal.pone.0305787)
Supplement: Attachment — Submitted filename: manuscript comments-1.docx [file pone.0305787.s003.docx]

The manuscript “Acquired hypothyroidism, iodine status and hearing impairment in adults: a pilot study” investigate the rate of hearing impairment in acquired hypothyroid patients. Manuscript designed well and consider for publication. The authors responded appropriately and incorporated all the information in the revised manuscript.
